# Skin Lesion Caused by ST398 and ST1 MRSA, Spain^1^

**DOI:** 10.3201/eid1601.090694

**Published:** 2010-01

**Authors:** Carmen Aspiroz, Carmen Lozano, Ana Vindel, Juan J. Lasarte, Myriam Zarazaga, Carmen Torres

**Affiliations:** Hospital Royo Villanova, Zaragoza, Spain (C. Aspiroz); Universidad de La Rioja, Logroño, Spain (C. Lozano, M. Zarazaga, C. Torres); Centro Nacional Microbiología, Madrid, Spain (A. Vindel); Centro Salud San Mateo de Gállego, Zaragoza (J.J. Lasarte)

**Keywords:** Methicillin-resistant Staphylococcus aureus, human infection, ST398, ST1, bacteria, Spain, letter

**To the Editor:** Human infections caused by methicillin-resistant *Staphylococcus aureus* (MRSA) sequence type 398 (ST398) have been emerging in recent years in Europe (*1**,**2*). Pigs represent a common reservoir of MRSA ST398, and working with these animals may constitute a risk factor for MRSA carriage and possible infection (*2**–**4*). We report a case of human infection caused by MRSA ST398 in Spain.

A 12-year-old girl living close to a pig farm, where her father and mother worked, sought treatment for a skin lesion on her chin. Two types of MRSA were recovered from the lesion, and it resolved after topical treatment with mupirocin over 10 days. MRSA isolates recovered were characterized by multilocus sequence typing (MLST) and by staphylococcal cassette chromosome (SCC) *mec*, *spa*, and *agr* typing as described (*3*). The presence of genes encoding Panton-Valentine leukocidin (PVL) (*lukF/lukS*), toxic shock syndrome toxin-1 (*tsst1*), and exfoliative-toxin A and B (*eta*, *etb*) was investigated by PCR (*2**,**3*). Antimicrobial susceptibility tests were carried out by using the VITEK-2 system (bioMérieux, Marcy l’Etoile, France), and *mecA,* *ermA*, *ermB*, *ermC*, *mrsA*, *tetK, tetL, tetM*, *ant(4′)-Ia*, *aph(3′)-III* , and *aph(2′)-aac(6′)* resistance genes were studied by PCR (*5*). *dfrK* gene detection was performed by using primers designed from the sequence FM207105 included in GenBank (*dfr*K-F 5′-GAGAATCCCAGAGGATTGGG; *dfr*K-R, 5′-CAAGAAGCTTTTCGCTCATAAA), and the obtained amplicons were sequenced for confirmation. Mutations in quinolone targets were determined by sequence analysis of *grlA* and *gyrA* genes (*6*). In addition, MRSA isolates were typed by pulsed-field gel electrophoresis (PFGE) (www.harmony-microbe.net/microtyping.htm).

One of the clinical MRSA isolates recovered from the lesion and typed as ST398-*spa-*t011 showed resistance to tetracycline, macrolides, and lincosamides and harbored 5 antimicrobial resistance genes. The second MRSA strain was typed as ST1-*spa*-t127 and showed a multiresistance phenotype with 11 resistance genes, as well as the Ser80Phe and Ser84Leu amino acid changes in GrlA and GyrA proteins, respectively.

To establish the MRSA nasal colonization status of the patient and of her relatives (father, mother, and brother, all of whom had contact with animals) and to elucidate the possible origin of these strains, we analyzed nasal swabs for MRSA recovery. Nasal samples were plated in oxacillin-resistant *S. aureus* agar media (Oxoid, Termofisher, UK), colistin nalidixic acid (bioMérieux), and blood agar media (Oxoid); colonies suggestive of *S. aureus* were initially selected, further identified, and characterized for bacterial typing and antimicrobial resistance mechanisms. Samples from all relatives and the patient showed that all 4 persons were MRSA nasal carriers presenting the following genetic lineages: patient girl, ST398-t108; mother, ST398-t108 and ST1-t127; father, ST398-t108; and brother, ST398-t011 (2 variants) (Table). MRSA t127 and t011 variants detected in the skin lesion of the patient were not found in her nasal sample but were found in the nasal samples of her mother and brother. Another study has been initiated to analyze the presence of MRSA in nasal swabs of swine on the farm.

**Table:** Characteristics of the 9 MRSA strains recovered in Spain from a patient’s lesion and from nasal samples obtained from patient’s family members*

Strain	Origin of sample	SCC*mec* type	MLST	*spa* type	*agr*	Antimicrobial resistance phenotype	Resistance genes detected	Amino acid change in:	
								GrlA	GyrA
C1570	Patient/ skin lesion	V	ST398	t011	I	OXA, FOX, TET, ERY, CLI, TEL	*mecA, tetK, ermA, ermC, msrA*	NP	NP
C1569	Patient/ skin lesion	II	ST1	t127	III	OXA, FOX, TET, ERY, CLI, TEL, GEN, TOB, KAN, CIP, LEV, SXT	*mecA, tetL, tetK, ermA, ermB, ermC, msrA, aph(2’)-acc(6’), ant(4´)-Ia, aph (3’), dfrK*	S80F	S84L
C1571	Patient/ nasal swab	V	ST398	t108	I	OXA, FOX, TET, ERY, CLI, TEL, CIP†, LEV†	*mecA, tetK, tetL, tetM, ermA, ermC, msrA*	Wild	Wild
C1577	Mother/ nasal swab	V	ST398	t108	I	OXA, FOX, TET, ERY, CLI, TEL, CIP†, LEV†	*mecA, tetK, tetM, ermA, ermC, msrA*	Wild	Wild
C1578	Mother/ nasal swab	II	ST1	t127	III	OXA, FOX, TET, ERY, CLI, TEL, GEN, TOB, KAN, CIP, LEV, SXT	*mecA, tetL, tetK, ermA, ermB, ermC, msrA, aph(2’)-acc(6’), ant(4´)-Ia, aph (3’), dfrK*	S80F	S84L
C1574	Brother/ nasal swab	V	ST398	t011	I	OXA, FOX, TET, ERY, CLI, TEL	*mecA, tetK, ermA, ermC, msrA*	NP	NP
C1576	Brother/ nasal swab	V	ST398	t011	I	OXA, FOX, TET, ERY, CLI, TEL, GEN, TOB, KAN, SXT	*mecA, tetL, tetK, ermA, ermB, ermC, msrA, aph(2’)-acc(6’), ant(4´)-Ia, aph (3’), dfrK*	NP	NP
C1572	Father/ nasal swab	V	ST398	t108	I	OXA, FOX, TET, ERY, CLI, TEL, CIP†, LEV†	*mecA, tetK, tetL, tetM, ermA, ermC, msrA*	Wild	Wild

*MRSA, methicillin resistant *Staphylococcus aureus*; SCC, staphylococcal cassette chromosome; MLST, multilocus sequence typing; ST, sequence type; OXA, oxacillin; FOX, cefoxitin; TET, tetracycline; ERY, erythromycin; CLI, clindamycin; TEL, telithromycin; NP, not performed; GEN, gentamicin; TOB, tobramycin; KAN, kanamycin; CIP, ciprofloxacin; LEV, levofloxacin; SXT, sulfamethoxazole-trimethoprim. †Iintermediate category for the indicated antimicrobial drug.

The ST1-t127 strains obtained from the skin lesion of the girl (C1569) and from the mother’s nasal sample (C1578) showed the same multiresistance phenotype and genotype (Table) and an indistinguishable PFGE pattern. This clonal type seems to be associated with community-acquired MRSA isolates circulating in Europe (*7*). The animal origin of this MRSA type and its possible transmission from horses and cows to humans has been suggested in previous reports (*8**,**9*).

In addition, all family members were colonized by MRSA ST398. All recovered nasal MRSA ST398 strains showed resistance to β-lactams, macrolides, lincosamide, and tetracycline, and 3 strains also showed diminished susceptibility to quinolones. Strain ST398 C1576, recovered from the brother, showed a multiresistance phenotype (Table).

The ST398 strains of our study were classified in the *spa*-types t011 and t108, 2 of the most frequently described types of the clonal complex 398 (*1**,**4*) and were untypeable by PFGE. Most MRSA ST398 strains in our study showed a resistance phenotype that included tetracycline, macrolides, and lincosamides. Tetracycline resistance is a common trait of ST398 and suggests that its use in veterinary medicine may have been implicated in selection of this resistance. The first reports, describing ST398 strains, indicated β-lactams and tetracycline as the unique resistance markers, which were susceptible to other antimicrobial agents. Nevertheless, reports of ST398 showing resistance to other antimicrobial drugs are increasing. One of the ST398 strains from the brother showed an uncommon multiresistance phenotype. Moreover, all the MRSA strains of this study were negative for all tested toxin genes, included PVL, although some ST398 PVL–positive strains have been occasionally described (*10*).

We report a skin lesion on the daughter of a pig farmer in Spain associated with ST398-t011 and ST1-t127 MRSA strains. The results obtained suggest the specific animal origin of these strains and subsequent transference among family members. Of special interest is the multiresistance phenotype of the clinical and nasal ST1-t127 MRSA clinical strains and of 1 nasal strain belonging to ST398 lineage. Nasal colonization by different ST398 genetic lineages and by other lineages of MRSA as ST1-t127 seems to be frequent in persons living in close proximity to farm animals. Dissemination of MRSA ST398 (and probably also MRSA ST1) in humans who have contact with farm animals, is an emerging problem in Spain.

## References

[1] Witte W, Strommenger B, Stanek C, Cuny C. Methicillin-resistant *Staphylococcus aureus* ST398 in humans and animals, Central Europe. Emerg Infect Dis. 2007;13:255–8. 10.3201/eid1302.060924 17479888 PMC2725865

[2] Lewis HC, Molbak M, Reese C, Aarestrup FM, Selchau M, Sorum M, Pigs as source of methicillin-resistant *Staphylococcus aureus* CC398 infections in humans, Denmark. Emerg Infect Dis. 2008;14:1383–9. 10.3201/eid1409.071576 18760004 PMC2603104

[3] Huijsdens XW, van Dijke BJ, Spalburg E, van Santen-Verheuvel MG, Heck ME, Pluister GN, Community-acquired MRSA and pig-farming. Ann Clin Microbiol Antimicrob. 2006;10:26. 10.1186/1476-0711-5-26 PMC1654169 17096847

[4] Voss A, Loeffen F, Bakker J, Klaassen C, Wulf M. Methicillin-resistant *Staphylococcus aureus* in pig farming. Emerg Infect Dis. 2005;11:1965–6. 16485492 10.3201/eid1112.050428 PMC3367632

[5] Domínguez E, Zarazaga M, Torres C. Antibiotic resistance in *Staphylococcus* isolates obtained from fecal samples of healthy children. J Clin Microbiol. 2002;40:2638–41. 10.1128/JCM.40.7.2638-2641.2002 12089295 PMC120538

[6] Schmitz FJ, Hofmann B, Hansen B, Scheuring S, Luckefahr M, Klootwijk M, Relationship between ciprofloxacin, ofloxacin, levofloxacin, sparfloxacin and moxifloxacin (BAY 12-8039) MICs and mutations in *grlA, grlB, gyrA* and *gyrB* in 116 unrelated clinical isolates of *Staphylococcus aureus.* J Antimicrob Chemother. 1998;41:481–4. 10.1093/jac/41.4.481 9598779

[7] Otter JA, French GL. The emergence of community-associated methicillin-resistant *Staphylococcus aureus* at a London teaching hospital, 2000–2006. Clin Microbiol Infect. 2008;14:670–6. 10.1111/j.1469-0691.2008.02017.x 18558939

[8] Juhász-Kaszanyitzky E, Jánosi S, Somogyi P, Dán Á, van der Graaf-van Bloois L, van Duijkeren E, MRSA transmission between cows and humans. Emerg Infect Dis. 2007;13:630–2. 10.3201/eid1304.060833 17553285 PMC2725960

[9] Cuny C, Strommenger B, Witte W, Stanek C. Clusters of infections in horses with MRSA ST1, ST254, and ST398 in a veterinary hospital. Microb Drug Resist. 2008;14:307–10. 10.1089/mdr.2008.0845 19025385

[10] Welinder-Olsson C, Florén-Johansson K, Larsson L, Oberg S, Karlsson L, Ahrén C. Infection with Panton-Valentine leukocidin-positive methicillin-resistant *Staphylococcus aureus* t034. Emerg Infect Dis. 2008;14:1271–2. 10.3201/eid1408.071427 18680653 PMC2600383

